# Cultural Stress Profiles: Describing Different Typologies of Migration Related and Cultural Stressors among Hispanic or Latino Youth

**DOI:** 10.1007/s10964-023-01784-9

**Published:** 2023-05-18

**Authors:** Ingrid Zeledon, Jennifer B. Unger, Alan Meca, Maria Duque, Ryan Lee, Daniel W. Soto, Trevor Pickering, Seth J. Schwartz

**Affiliations:** 1 University of Southern California Keck School of Medicine, Los Angeles, CA USA; 2 University of Texas at San Antonio, San Antonio, TX USA; 3 University of Texas at Austin, Austin, TX USA

**Keywords:** Cultural Stressors, Hispanic/Latino Youth, Latent Profile Analysis, Depression, Immigrant

## Abstract

Youth of immigrant origin vary across their families’ migration history (e.g., country of heritage, reasons for migration, etc.) and in the communities in which they reside. As such, these youth are often faced with different cultural and immigrant stressors. Although prior research documented the detrimental impact of cultural and immigrant stressors, variable-centered approaches fail to account for the fact that these stressors often co-occur. Addressing this gap, the current study identified typologies of cultural stressors in Hispanic/Latino adolescents using latent profile analysis. Cultural stress profiles were derived using socio-political stress, language brokering, in-group identity threats, and within-group discrimination as indicators. The study was conducted in two sites (Los Angeles and Miami; total *N* = 306) during Spring and Summer 2020. A four-profile solution was identified: Low Cultural Stress (*n* = 94, 30.7%), Sociopolitical and Language Brokering Stress (*n* = 147, 48%), Sociopolitical and In-group Identity Threat Stress (*n* = 48, 15.7%), and Higher Stress (*n* = 17, 5.6%). Results indicate that profiles with stress were characterized by worse mental health symptoms, reporting higher means of depression, stress, and lower self-esteem, as well as by higher heritage cultural orientation compared to the low stress profile. Interventions designed to mitigate the deleterious effects of cultural stressors would benefit from adopting an individualized, tailored approach that addresses youth’s stress profile membership.

## Introduction

Adolescence is marked by rapid changes in brain development, identity formation, and socially ascribed roles (Griffin, 2017). These transitions often increase adolescents’ susceptibility to environmental influences and sensitivity to stressors, which in turn can place youth at risk for substance use, problematic behaviors, and mental health challenges (Johnson et al., 2019). In addition to commonly experienced adolescent stressors, Hispanic/Latino^1^ youth in the United States (U.S.) contend with a variety of unique stressors such as discrimination and immigration-related stressors, which may compound the detrimental effects of other stressors (Torres & Young, 2016). Systematic reviews have identified strong positive associations between cultural stressors and mental health outcomes (e.g., depressive symptoms, anxiety, self-esteem) (Choy et al., 2021). However, studies have not determined whether there are specific subgroups of Hispanic/Latino adolescents who experience different combinations of cultural stressors. Understanding typologies of cultural stress can assist in the creation of tailored interventions for specific subgroups of adolescents. To address this gap, the current study was designed to identify cultural risk profiles and to examine the association of these profiles across three mental health indicators (i.e., depressive symptoms, self-esteem, and adolescent stress) and three cultural assets of interest (i.e., U.S. orientation, heritage orientation, and ethnic identity).

### Transnational Theory of Cultural Stress

The Transnational Theory of Cultural Stress is a framework conceptualizing the pathway through which cultural stress influences health outcomes (Salas-Wright et al., 2020). Cultural stress refers to the unique stressors experienced by youth navigating multiple cultural streams and negotiating cultural identity, traditions, and values (Schwartz et al., 2015). Similar to other stress process theories (e.g., Simmons & Nelson, 2012), the Transnational Theory of Cultural Stress posits that stress responses can result in experiencing either distress or eustress (i.e., a stressor that contributes to well-being, such as increased motivation or improved cognitive functioning). The response experienced by an individual confronting a stressor is dependent upon their cognitive appraisal, or evaluation of the impact the stressor has on their wellbeing and their assessment of the resources available to manage the stressor (Lazarus & Folkman, 1984). Coping responses represent intentional attempts to regulate emotions, thoughts, behaviors, or physiology to mitigate stress and its sequelae (Compas et al., 2014). Adaptive coping mechanisms can contribute to an individual’s resiliency, or their ability to positively adapt despite adversity or trauma (Compas, 2013).

For this reason, the Transnational Theory of Cultural Stress theory is embedded within a risk and resiliency lens and posits that cultural and migration related stressors create tensions among parents and their children, which then compromise family functioning and increase risks for maladaptive health behaviors. Cultural stressors are often operationalized as risk factors – characteristics associated with poor mental or health outcomes – and most of the cultural stressors that have been studied, aside from some studies on language brokering, are associated with risky behaviors and compromised mental health symptoms. Moreover, this framework emphasizes that cultural stress is additive, with higher levels of stress and more sources of stress being associated with adverse mental health outcomes (McCord et al., 2019), highlighting the need for research to attend to multiple cultural stressors as well as the ways in which multiple cultural stressors can impact mental health.

### Key Cultural Stressors Experienced by Hispanic/Latino Youth

Conceptualizations of cultural and immigration-related stressors have varied widely, ranging from acculturative stress and parent-child acculturation gaps, bicultural stress, discrimination, negative context of reception, and adverse childhood experiences (e.g. unrest or violence in one’s country of origin, dangerous journey, and instability resulting from immigration) (Zeledon et al., 2023). Recent research on immigrant-origin youth in the U.S. (e.g., Vos et al., 2021) documented four key self-identified cultural stressors: (1) political stress (i.e., impact of anti-immigrant rhetoric, fear of deportation, or termination of immigrant-friendly policies such as the Deferred Action for Childhood Arrivals (DACA) program,), (2) Language Brokering (i.e., responsibility for translating between English and Spanish, generally for parents or other close family members), (3) in-group identity threats (i.e., being considered ‘too American’ by other Hispanics/Latinos), and (4) within group discrimination (i.e., being bullied or insulted by other Hispanics/Latinos). The present study focuses on examining the impact of these four stressors on mental health symptoms.

These four domains of stress have not only been highlighted by youth themselves (Vos et al., 2021), but have been previously identified in the literature to represent important cultural stressors. For example, in terms of political stress, negative sociopolitical climate has been associated with increased fear, worry, and perception of decreased opportunities for advancement (Roche et al., 2021). The deleterious effects (e.g., excessive worry and depressive symptoms) of a negative sociopolitical climate are more pronounced for individuals who are not citizens or have family members who are undocumented (Roche et al., 2020). A negative sociopolitical climate may also moderate the relationship between acculturation and stress-related health problems (higher acculturation is correlated with more stress-related health problems), which may potentially be attributed to increased exposure to discrimination in regions that are unwelcoming toward migrants (Almeida et al., 2016). Moreover, changes in immigration policies, such as the proposed discontinuation of Deferred Action for Childhood Arrivals (DACA), have been hypothesized to exacerbate mental health issues due to the uncertainty that such legislative changes triggers (Cadenas et al., 2022).

Language brokering, or the practice of children or adolescents serving as translators or interpreters on behalf of their parents, can have both positive (e.g., empathy, bilingual ability, and increased family pride) and negative (e.g., stress relating to accuracy of translation, time demands, burden of translating complex or negative information across different contexts) effects on mental health (Kam & Lazarevic, 2014). Adolescents who engage in language brokering report higher levels of depression when compared to non-language brokers, and this high language brokering stress is associated with a higher cortisol secretion (Kim et al., 2022). Although language brokering efficacy and language brokering norms may buffer the stress experienced during language brokering, experiencing discrimination can be associated with greater language brokering burden (Lazarevic et al., 2022).

Within-group identity threats and within-group discrimination have been included under the heading of intragroup marginalization, or perceived rejection by people within one’s heritage-cultural group for deviating too far from heritage-cultural norms (Castillo, 2009). Individuals can experience, therefore, marginalization in either direction, either for being “too Hispanic” or “too American.” It is theorized that intragroup marginalization subverts an individual’s sense of belonging and self-identity, which are pivotal for well-being. (Zhen-Duan et al., 2011). As such, intragroup marginalization is often associated with higher depressive symptoms and lower self-esteem (Thornhill et al., 2021).

### Demographic Factors and their Influence on Cultural Stressors

Studies have consistently found that males and females respond differently to stressful conditions, with females experiencing internalizing symptoms (e.g., anxiety or depression) and males exhibiting more externalizing symptoms (e.g., aggressive behavior, drinking, etc.) (Hill & Needham, 2013). Such gender differences have also been found in cultural stress studies among Hispanic/Latino youth, plausibly due to gender norms, where externalizing behaviors are viewed as especially unacceptable in females (Castilla-Puentes et al., 2022). Taken together, these findings highlight the need for researchers to analyze sex differences in the examination of cultural stressors.

At the same time, as theorized by the Transnational Theory of Cultural Stress (Salas-Wright et al., 2021), the experiences and impact of cultural stressors are likely to differ as a function of context, as well as broader social-cultural historical forces. Indeed, in a recent study focused on political stress before and after the 2020 U.S. presidential election, participants’ political stress increased post-election in Miami, where the local political context was most discrepant in comparison to the national post-election results, but not in Los Angeles, highlighting that how youth’s perceive socio-political climate is complex and informed by their local political context (Montero-Zamora et al., 2023b). These findings emphasize that, despite both being considered cultural enclaves, Miami, and Los Angeles (sites where the present study was also conducted) differ greatly in their socio-political context. In terms of country of origin, the majority of Latinos in Miami are Cubans who, unlike other Latinos, have had an automatic path to citizenship in the US and, therefore, are less impacted by political issues or national sentiments around immigration (Fandl, 2017). It is estimated that 58% of Cubans in the U.S. are registered Republican voters in comparison to 32% of non-Cuban Hispanics (Krogstad, 2021). By contrast, most Latinos in Los Angeles are either of Mexican or Central American heritage, many are undocumented, and for those who have family near the southern border, stricter border enforcement would make it difficult to travel between the U.S. and Mexico (Passel & Cohn, 2020). Moreover, these findings highlight the prevalence of socio-political stress that were tied to the broader political climate during the 2020 U.S. presidential election, which was when data for this current project was collected.

Nativity status, either of the parents or the youth, is often a proxy for documentation status itself or exposure to documentation issues within the family, as it might be indicative of an early family migration event. Being documented bestows privileges that impact day to day functioning, such as having a driver’s license in some U.S. states, or economic opportunities, such as having a work permit and better employment prospects (Enriquez et al., 2018). Having a mixed documentation status family or having ties to individuals who are undocumented may contribute to feelings of emotional insecurity, uncertainty, or hypervigilance, which are associated with symptoms of anxiety and depression (Rodriguez, 2016).

### Protective Effects of Cultural Assets

Rooted within a risk and resilience framework, the Transnational Theory of Cultural Stress emphasizes the importance of promotive and protective factors. For example, family functioning, or the quality of relationships among family members, is considered as a promotive factor (i.e., a characteristic that is associated with positive outcomes irrespective of risk exposure), and compromised family functioning serves as an the underlying mechanism through which cultural stress impacts health outcomes (Lorenzo-Blanco et al., 2012). Additionally, cultural assets, such as ethnic and U.S. identity, may serve as protective factors – aspects of the individual or environment that offset the effects of risk factors (Davis et al., 2021).

The sections above summarize findings on cultural stressors as risk factors across multiple mental health indicators (e.g., depression and anxiety). This current section defines and provides a summary of findings on the most studied cultural assets: ethnic identity, heritage orientation, and destination-cultural orientation. Ethnic identity refers to a positive sense of self derived from being a member of a heritage group, as well as to the positive feelings that this attachment elicits (Rivas-Drake et al., 2021). Ethnic identity has been identified as predictive of self-esteem and other forms of well-being (Smith & Silva, 2011). Ethnic identity has also been found to buffer the negative effects of discrimination, wherein identifying with one’s cultural heritage provides social support and belonging that offset the effects of outgroup rejection (Cobb et al., 2019).

For the purposes of the present study, among immigrant-origin youth, heritage orientation refers to retaining the practices of one’s heritage country, irrespective of immigrant generation (i.e., foreign born or U.S. born to foreign born parents) (Schwartz et al., 2010). Across many health indicators, heritage orientation has been found to be protective. Research suggests that specific cultural values (e.g., familism), as well as enhanced social support, contribute to explaining the protective effect of heritage orientation (Unger et al., 2002). U.S. orientation, in this study, is defined as engagement with U.S. cultural practices (e.g., Americanized peer affiliations, listening to English language music, and speaking English). U.S. orientation may also be protective against substance use and other negative outcomes (Schwartz et al., 2014). Biculturalism, referring to high levels of both heritage and U.S. orientation, may be especially protective and has been associated with higher self-esteem ethnic identity, development, and with lower levels of mental health distress (lower anxiety and depressive symptoms) and of substance use (Perreira et al., 2019). Biculturalism provides adaptive skills in that individuals can draw on resources from both cultures to cope with stressors (Smokowski & Bacallao, 2011).

## The Current Study

Although a substantive body of research has examined the detrimental effects of cultural stressors on mental health, this work has not considered the fact that cultural stressors naturally co-occur to varying extents among different subgroups of youth. As such, there is a key need to identify unique configurations of cultural stressors that can impair mental health and key protective factors. Addressing this gap, four salient indicators of cultural stress (i.e., sociopolitical climate, language brokering, in-group identity threat, and within-group discrimination) were used to identify and describe distinct youth cultural stressor profiles, identify demographic covariates of profile membership, and analyze differences in mental health indicators and cultural assets across profile membership. Although no specific hypotheses were advanced regarding the specific number of or structure of the unique configurations of cultural stressors, given the prevalence of socio-political turmoil at the time of data collection (spring/summer 2020), it was expected that high mean for sociopolitical climate stress to emerge across profiles (Hypothesis 1). Additionally, it was expected that sex differences would emerge across profiles, with females overrepresented within profiles defined by higher mean stress scores (Hypothesis 2). Finally, it was expected that differences would emerge in mental health outcomes (i.e., depression, self-esteem, adolescent stress) and cultural assets (i.e., ethnic identity, U.S. orientation, heritage orientation) across profiles with those defined by higher levels of cultural stress, associated with adverse mental health outcomes (Hypothesis 3). By furthering the understanding of how unique configurations of cultural stressors are experienced among Hispanic/Latino youth, the current study may provide insights into tailored interventions to increase resiliency and reduce appraisals of stress.

## Methods

### Procedures

Data for this study were collected from a multi-phase mixed methods project. First, qualitative interviews (6 focus groups, *N* = 34; Vos et al., 2021) were conducted to identify cultural stress themes. Then, an expert panel was convened to develop items. A baseline survey was collected, consisting of these items as well as additional items adapted from existing validated scales, to create a holistic measure of cultural stress for Hispanic youth. This effort yielded the Multidimensional Inventory of Cultural Stress, subscales from which are used in this study as indicators in the latent profile analysis. Participants were recruited through schools in Los Angeles and Miami-Dade Counties, and selection criteria included: (1) either being born, or having at least one parent who was born, in a Spanish speaking country (Cuba, the Dominican Republic, Mexico, Central America, or South America); (2) self-reporting or identifying as Latino/Hispanic, and (3) speaking English or Spanish fluently. Data were collected in the Spring and Summer of 2020 from students attending one of three high schools (two high schools in Los Angeles and one high school in Miami). Parents and youth were recruited through phone calls, text messages, and emails. Due to COVID-19, study details, assent, consent, and survey were communicated virtually through RedCap and Qualtrics. Recruitment was targeted, such that only youth that met inclusion criteria were asked to participate in the study. Participants received a $15 gift card as an incentive for participation. Study procedures were approved by the Institutional Review Boards at the participating universities.

### Participants

Survey responses from 306 Hispanic/Latino adolescents (18.9% in 9^th^ grade, 50.2% in 10^th^ grade, and 30.3% in 11^th^ grade) with a mean age of 15.30 (*SD* = 0.76, range 14–17 years) were included in the present analyses. Participants were predominantly second generation (U.S. born to at least one foreign born parent, 79.55%), with the remainder being first generation immigrants. The majority of participants and/or their parents were Mexican (56.3%), Cuban (26.5%), or Central American (i.e., Salvadoran, Honduran, Nicaraguan, and Guatemalan; 11.2%). Table 1 provides demographic information for the overall sample.

**Table 1:** Participant demographic information

Variable	Overall sample% (*N*)	Profile 1: Low	Profile 2: Social climate & language brokering	Profile 3: Social climate & in-group identity threat	Profile 4: High	χ^2^ *P*-value
	*N* = 306 (%)	*n* = 94 (30.7%)	*n* = 147 (48.0%)	*n* = 48 (15.7%)	*n* = 17 (5.6%)	
Site						
Miami	35.95% (*N* = 110)	52 (55.32%)	39 (26.53%)	15 (31.25%)	4 (23.53%)	<0.001
Los Angeles	64.05% (*N* = 196)	42 (44.68%)	108 (73.47%)	33 (68.75%)	13 (76.47)	
Sex	–	–	–	–	–	
Female	60.13% (*N* = 184)	53 (56.38%)	95 (64.63%)	36 (75.00%)	14 (82.35%)	0.96
Male	39.22% (*N* = 120)	39 (41.49%)	52 (35.37%)	12 (25.00%)	3 (17.65%)	
Missing	0.65% (*N* = 2)	2 (2.13%)				
Birth	–	–	–	–	–	0.05
Foreign Born	20.59% (*N* = 63)	29 (30.85%)	27 (18.37%)	2 (4.17%)	4 (23.53%)	
Born in U.S.	79.41% (*N* = 243)	65 (69.15%)	120 (81.63%)	46 (95.83%)	13 (76.47%)	
Parental Birth	–	–	–	–	–	0.59
Both Foreign Born	75.16% (*N* = 230)	60 (63.83%)	139 (94.56%)	17 (35.42%)	14 (82.35%)	
1 + U.S. Born Parent	24.51% (*N* = 75)	34 (36.17%)	8 (5.44%)	30 (62.50%)	3 (17.65%)	
*Missing*	0.33% (*N* = 1)			1 (2.08%)		
	**Mean (SD)**	**Mean (SD)**	**Mean (SD)**	**Mean (SD)**	**Mean (SD)**	
Age	15.30 (0.76)	15.32 (0.70)	15.32 (0.83)	15.31 (0.69)	15.06 (0.77)	0.64
SES	2.24 (1.10)	0.04 (0.94)	2.37 (1.24)	2.19 (0.96)	2.44 (0.91)	<0.01
Depression	2.20 (0.66)	1.97 (0.07)	2.44 (0.12)	2.23 (0.06)	2.50 (0.16)	<0.01
Self-Esteem	3.32 (0.77)	3.62 (0.10)	3.17 (0.12)	3.21 (0.08)	3.06 (0.13)	<0.01
Adolescent Stress	2.85 (0.81)	2.32 (0.10)	3.34 (0.11)	2.97 (0.07)	3.40 (0.21)	<0.001
American Orientation	4.19 (0.69)	4.20 (0.10)	4.40 (0.10)	4.20 (0.06)	3.84 (0.21)	0.10
Heritage Orientation	4.10 (0.80)	3.80 (0.10)	3.63 (0.13)	4.43 (0.06)	4.33 (0.20)	<0.001
Ethnic Pride	3.50 (0.65)	3.11 (0.10)	3.73 (0.10)	3.72 (0.06)	3.93 (0.19)	<0.001

### Measures

#### Cultural stress

The Multidimensional Inventory of Cultural Stress is an adolescent cultural and immigration related stress measure consisting of four subscales: (1) Negative Sociopolitical Climate (6 items, α = 0.90), (2) In-group Identity Threats (3 items, α = 0.88), (3) Language Brokering (3 items, α = 0.77), and (4) Within-Group Discrimination (2 items, α = 0.77). Participants’ responses were recorded on a 5-point Likert scale ranging from 1 (strongly disagree) to 5 (strongly agree). Negative sociopolitical climate assesses the level of distress felt around policies (e.g., “I am worried what the end of Deferred Action for Childhood Arrivals (DACA) and other protections would do to my family and friends” and “I am worried that people I love will be deported”) and the political environment (e.g., “With the current political situation, I have felt more fearful”). In-group identity threats refer to distress felt by participants when other Hispanic individuals question their heritage identity (e.g., “Other Hispanics/Latinos often tell me I’m too American/Anglo/Gringo” and “Other Hispanics/Latinos criticize me for not speaking Spanish well”). The language brokering subscale, reflecting a well-documented stressor in the literature (Weisskirch, 2017), assesses pressures stemming from acting as a translator for close family members (e.g., “I feel like I’m responsible for helping my parents understand things in English”). Lastly, within-group discrimination captures perceptions that other Hispanic individuals believe that one is too strongly ethnically identified (e.g., “Other Hispanics/Latinos often tell me I’m too Hispanic/Latino or not American enough” and “Other Hispanics/Latinos have called me insulting names (like ref (refugee), arrow-thrower)”). In the present study, the four subscales of the MICS were used as primary indicators of profile membership.

#### Depressive symptoms

The 10-item Boston form of the Center for Epidemiologic Studies Depression Scale (CESD-10) Andresen et al., 1994) was used to assess depressive symptoms. Each item (e.g. “I felt sad”) is rated on a 4-point Likert scale (α = 0.87) ranging from 0 (rarely or none of the time) to 3 (almost all the time). Scores were calculated as the sum of all item responses.

#### Ethnic identity

The Multigroup Ethnic Identity Measure (MEIM; Phinney, 1992) is a 12-item scale (α = 0.91) assessing sense of belonging, ethnic identity, and involvement. Items are rated on a 4-point Likert scale ranging from 1 (strongly disagree) to 4 (strongly agree) and analyzed using the total sum of the items, with higher scores indicating a stronger sense of ethnic identity.

#### Self-esteem

Self-esteem was measured using the Rosenberg Self-Esteem Scale (Rosenberg, 1979), which consists of 10 items (α = 0.86). Items are rated on a 4-point Likert scale ranging from 1 (strongly agree) to 4 (strongly disagree). Five items are reverse coded (e.g., “I certainly feel useless at times”). Higher scale scores indicate higher self-esteem. Scores were calculated as the total sum of all items.

#### Adolescent stress

The Adolescent Stress Questionnaire (ASQ-S*)* measures normative stressors specific to adolescence as a developmental period (Anniko et al., 2018). The short version utilized in this study is composed of the sum of 27 items assessing the following stressors: home life, school performance, school attendance, romantic relationships, peer pressure, teacher interaction, future uncertainty, school/leisure conflict, financial pressures, and emerging adult responsibilities (α = 0.94). All items are rated on a 5-point Likert scale ranging from 1 (not stressful at all) to (5) (very stressful*)*. Scores were calculated as the sum of all item responses.

#### U.S and heritage orientation

U.S. and heritage cultural orientations were measured as independent subscales of the Bicultural Involvement Questionnaire (Szapocznik et al., 1980). Each subscale contains 12 questions that mirror each other and measure involvement separately in the U.S. and heritage cultures (e.g., How comfortable do you feel speaking [Spanish or English] at home?). The items are rated on a 5-point Likert scale and measured as a mean of the items for each subscale (U.S. orientation, α = 0.90; heritage orientation, α = 0.92).

### Statistical Analysis Plan

Latent Profile Analysis extracts different patterned profiles (as a latent categorical variable) from a set of continuous variables (indicators) of interest (Spurk et al., 2020). This categorical latent modeling approach assumes that a given population contains subpopulations with configural differences across the indicators of interest and varying class membership probabilities (Howard & Hoffman, 2018). Socio-political stress, language brokering, in-group identity threats, and within-group discrimination were used as indicators for the latent profiles. These indicators were screened for outliers through visual inspection of distributions to ensure that profile enumeration was not biased by extreme cases or implausible values. After initial data inspection, a series of latent profile models were generated using full information maximum likelihood (FIML) estimation. To ensure the profile solutions did not converge at a local maximum, 1000 random sets of starting values were utilized. Data management and clean-up were conducted in SAS software version 9.4. and Mplus Version 8.3 (Muthén & Muthén, 2018) was utilized to conduct the latent profile analysis.

After removing outliers, which is required within cluster analytic procedures, the effective sample consisted of 306 adolescents. Levels of missingness in the data were low, including some demographic information (reported in Table 1), but none of the variables used in the latent profile analysis were missing for any individual. Comparison of model fit statistics (Log Likelihood, Akaike information criterion (AIC), Bayesian information criterion (BIC), Lo-Mendell-Rubin Likelihood Ratio test (LRT), and entropy) was performed to determine the optimal number of profiles in terms of the lowest Akaike Information Criterion (AIC) and Bayesian Information Criterion (BIC) values, entropy values of .80 of greater, and Vuong-Lo-Mendell-Rubin likelihood ratio test (LRT) p-values < 0.05 (Ferguson et al., 2020). The one-step manual BCH method was utilized to analyze associations across variables of interest and profiles by performing Wald Chi-square tests on each outcome variable between all pairs of profiles (Asparouhov & Muthén, 2021). The BCH method creates weighted class membership probabilities that are used to determine class membership and to avoid profile misclassification issues that can occur when individuals are assigned to their most likely profiles. Commonly reported demographics of interest (i.e. sex, nativity status, and parental nativity status), along with site, were included as exogenous independent variables. Covariates were selected based on theoretical relevance to cultural stressors and a multicollinearity assessment to select non-multicollinear covariates (pairwise correlations >0.7 and VIF > 5) (Vatcheva & Lee, 2016). Based on these criteria, site (Miami versus Los Angeles), sex, youth nativity status, and parental nativity status were selected as covariates vis-à-vis profile membership. Covariates are reported as odds ratios, where one profile is selected as the reference group and the other profiles are compared against the reference group vis-à-vis each of the covariates. For odds ratios, the null value is 1, where values below 1 indicate a negative relationship with class membership and values above 1 indicate a positive relationship with class membership.

In addition, pairwise comparison of profiles across outcomes of interest (i.e., depressive symptoms, self-esteem, ethnic pride, cultural orientation, and adolescent stress) were conducted. Consistent with the BCH 1-step approach to latent profile analysis (Asparouhov & Muthén, 2021), these comparisons are reported as Wald chi-square values. A Bonferroni correction (α = 0.008) was implemented to account for multiple comparisons across profiles (DeMaris, 2004).

## Results

### Profile Solution Selection and Demographic Characteristics

Model fit statistics were compared across the models, in conjunction with entropy and theoretical significance, to yield a four-profile solution. For each class solution considered (i.e., 2-class, 3-class, 4-class), profile indicators were examined to determine whether these classes were similar to any from the 4-class solution (See Table 2). The four-profile solution was associated with the lowest AIC, BIC, and SSABIC values, and the Lo-Mendell-Rubin test favored a 4-profile solution in favor of a 3-profile solution (*p* = 0.049) but did not favor the 5-profile solution in favor of the 4-profile solution (*p* = 0.190). The entropy values were similar across all solutions (>0.80). Therefore, the 4-profile solution was selected as the championed model. None of the profiles had membership probabilities less than 5% of the full sample (see Nylund et al. (2007), for a review of criteria for selecting a latent profile solution), with the high stress profile associated with the lowest probability of membership (5.6%).

**Table 2:** Model Fit Indices for one to five profile model LPA Solutions

Fit statistics	Number of profiles			
	2	3	4	5
LL (No. of Parameters)	−1903.805 (13)	−11860.844 (18)	−**1827.544 (23)**	−1811.666 (28)
AIC	3833.609	3757.689	**3701.087**	3679.333
BIC	3882.016	3824.713	**3786.730**	3783.593
SSABIC	3840.786	1767.626	**3713.784**	3694.70
Lo-Mendell-Rubin (LMR)	1 vs 2	2 vs 3	**3 vs 4**	4 vs 5
LMR Probability	<0.001	0.0078	**0.0492**	0.1948
Entropy	0.830	0.863	**0.834**	0.843
Group Size (*N*, %) P1: Low	107 (35.0%)	107 (35.0%)	94 (30.7%)	–
P2: Social Climate & Language Brokering	199 (65.0%)	179 (58.5%)	147 (48.0%)	–
P3: Social Climate & In-group identity threat	–	–	48 (15.7%)	–
P4: High	–	20 (6.5%)	17 (5.6%)	–

The bold values are used to identify the selected number of classes in the final model. The model fit indexes do not have associated *p* values.

**Fig. 1:**
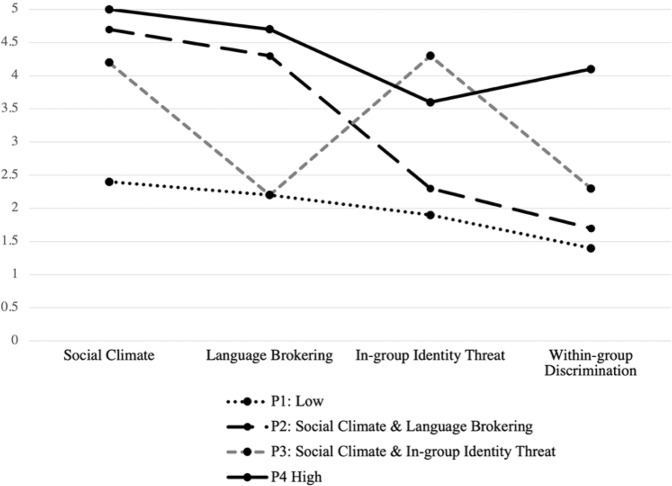
Means of indicators by latent profile

Figure 1 illustrates the mean for each indicator variable across the four identified profiles, and Table 1 provides sample demographics by the most likely profile membership. Profile 1, labeled as Low Cultural Stress (*n* = 94, 30.7% of the sample), and was characterized by low mean scores (under 2.5) across all four cultural stress subscales. Participants in this profile were predominantly female (61.29%) and U.S. born (79.55%) with foreign born parents (74.75%). Individuals in Profile 2 (*n* = 48, 15.7% of the sample), labeled as Sociopolitical and Language Brokering Stress, reported high levels of negative sociopolitical and language brokering stress; youth in this profile were predominantly female (61.64%) and U.S. born (80.14%) with foreign born parents (76.55%). Profile 3 (*n* = 147, 48% of the sample), labeled as Sociopolitical and In-Group Identity Threat Stress, was characterized by high levels of negative sociopolitical and in-group identity threat stressors; 58.33% were female with 87.5% foreign born, and with 72.92% reporting foreign born parents. Lastly, Profile 4 (*n* = 17, 5.6% of the sample), labeled as Higher Cultural Stress, included participants with high mean scores (over 3.5) across all four subscales; 56.25% of participants were female, with 94.12% reporting as U.S. born with 62.5% having foreign born parents.

Variables associated with profile membership were analyzed across site, nativity status, sex, and parental nativity status (see Table 3). Site effects were present; Sociopolitical and Language Brokering (profile 2; *OR* = 4.98, *p* < 0.001) and Higher stress (profile 4; OR = 8.50, *p* < 0.01) had a higher proportion of participants from Los Angeles than from Miami when compared to the low stress profile. Being US-born was a significant predictor of membership in the sociopolitical climate and in-group identity threats (OR = 8.50, *p* < 0.01) compared to the low stress profile. Sex emerged as a statistically significant correlate across all profiles (Sociopolitical and Language Brokering, OR = 3.60, *p* < 0.001 l; Sociopolitical and In-group Identity Threat; OR = 3.78, *p* < 0.01, Higher stress; OR = 8.86, *p* < 0.01). Lastly, for profile High (profile 4; OR = 5.71, *p* <0.05) and Sociopolitical and Language Brokering (profile 2; OR = 17.78, *p* < 0.001), parents’ nativity (having foreign born parents) emerged as a significant predictor of profile membership when compared to the low stress profile.

**Table 3:** Variables associated with profile membership

	2 vs. 1	3 vs. 1	4 vs. 1
Site (Los Angeles)	4.98 (2.41, 10.33)***	1.36 (0.58, 3.19)	8.50 (1.76, 41.09)**
Birth (US Born)	1.42 (0.64, 3.16)	7.24 (1.46, 35.92)*	0.65 (0.13, 3.26)
Sex (Female)	3.60 (1.92, 6.72)***	3.78 (1.68, 8.50)**	8.86 (2.27, 34.61)**
Parental Nativity (Foreign Born)	17.78 (7.23, 43.71)***	0.60 (0.27, 1.35)	5.71 (1.37, 23.90)*

OR (95 % CI) ***<0.001; **<0.01; *<0.05; Bonferroni correction: α = 0.008.

Across the predictor variables, only parental nativity status was a constructed categorical variable. This variable was categorized as either one or both parents foreign born compared to neither parent foreign born. It could have been categorized as both parents foreign born compared to one or neither parent foreign born, but this classification was not performed due to a small number of individuals with both parents foreign born. No other variables in the analysis required classification decisions by the authors.

### Differences in Mental Health Outcomes and Cultural Assets

**Fig. 2:**
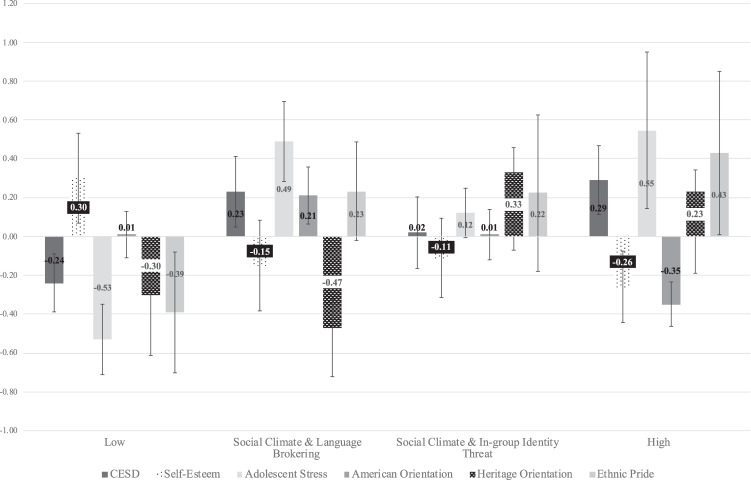
Mean levels across variables of interest by profile membership

The Wald chi-square test for equality of means yielded differences among the profiles (See Fig. 2). Compared to the Low Cultural Stress profile, the other three profiles were characterized by worse mental health indicators, reporting higher means of depression symptoms, adolescent stress, and lower self-esteem. However, Sociopolitical and Language Brokering profile was associated with a higher adolescent stress mean compared to the Climate and In-group Identity Threat profile. In terms of cultural assets, the other three profiles were associated with higher ethnic identity compared to the low stress profile. There were no differences in mean levels of U.S. orientation among the profiles. However, there were significant differences in heritage orientation, with the Sociopolitical and In-group Identity Threat and high profiles associated with higher mean heritage orientation when compared to the low stress profile. The Low and Sociopolitical and Language Brokering profiles scored similarly on heritage orientation. Both the Sociopolitical and In-group Identity Threat and Higher Stress profiles had significantly higher heritage orientation means compared to the sociopolitical and language brokering profile.

## Discussion

Hispanic/Latino youth are confronted with multiple migration-related and cultural stressors, at times simultaneously, in their daily interactions. Although there is a vast literature on the associations of cultural stressors on health, more studies are needed to understand how cultural stressors are experienced among subgroups of youth. The present study was designed to identify cultural stress profiles among first and second generation Hispanic/Latino youth and to examine how mental health indicators and cultural assets would differ across these profiles. The latent profile analysis yielded four typologies of cultural risk: (1) Low, (2) Sociopolitical and Language Brokering, (3) Sociopolitical and In-group Identity Threats, and (4) Higher stress. As hypothesized, findings suggest that profiles characterized by elevated cultural stress were associated with more maladaptive mental health indicators (higher depressive symptomology, higher adolescent stress, and lower self-esteem). Across the cultural assets, which were theorized as resiliency factors, profiles characterized by higher stress were associated with higher ethnic pride scores compared to the low stress profile, whereas heritage cultural orientation was higher in sociopolitical and in-group identity threat and high profiles compared to the low or sociopolitical and language brokering profiles. Together, these findings suggest that cultural risk profiles do vary among Hispanic/Latino youth, that profiles characterized by elevated cultural stressors are associated with poor mental health outcomes, and that levels of cultural assets also vary across the cultural stress profiles.

### Specific Cultural Stress Profiles

This study identified four cultural stress profiles (i.e., Low, Sociopolitical Climate and Language Brokering, Sociopolitical and In-group Identity Threat, and Higher stress). As predicted, high levels of sociopolitical climate emerged across all the extracted profiles with the exception of the Low stress profile. This finding highlights the importance of policies and structures that impact immigration issues for Hispanic/Latino youth. At the time of data collection, political discourse and immigration issues were on the forefront of news and discussion throughout the U.S., and this sociopolitical climate could have contributed to the prominence of sociopolitical stressors (Díaz McConnell, 2022). Therefore, socio-political stressors are likely to vary across time depending on the political discourse, which can aggravate or attenuate its effects on mental distress (Montero-Zamora et al., 2023a).

It is likely that members of Sociopolitical and Language Brokering in comparison to Sociopolitical and In-Group Identity Threat are either bilingual speakers with a high burden (e.g., high frequency of brokering and translating complex information) or are English dominant speakers with low perceived brokering efficacy – which are identified characteristics predictive of language brokering stress in the literature (Weisskirch, 2017). Because the majority of the sample is U.S. born, it is less likely that language brokering stress in this sample stems from difficulties with the English language. However, difficulties in translating could potentially arise from explaining complex concepts for which they lack the vocabulary or understanding.

It is also significant that in-group identity threats, but not within-group discrimination, emerged as a cultural stress profile in the present results. This finding could signal that, in relation to ingroup marginalization, in-group identity threats are more insidious to mental health and are perceived and reported more severely than in-group discrimination. Plausibly, in-group identity threats may be especially deleterious due to decreased sense of belonging to one’s ethnic or cultural group – where such lack of belonging may interfere with youth’s identity development process (Meca et al., 2022). Indeed, self-verification theory (Swann & Buhrmester, 2012) holds that group identifications are more secure, and are more facilitative of well-being, when they are validated by other members of the group in question.

The Higher stress profile included participants who experience high levels of stress across all subscales, experiencing alienation from both within-group (high levels of in-group discrimination and in-group identity threats) and external (i.e., an unwelcoming sociopolitical environment) forces. The simultaneous experience of ingroup identity threats and outgroup discrimination for a subgroup that is slightly higher heritage oriented than U.S. oriented could indicate higher bicultural stress, which was not measured in the present study. Highly culturally stressed youth may often navigate contexts where they are less acculturated than their peers but might nonetheless be perceived by their family or immediate community as losing aspects of their cultural heritage. Because the items used in the present study do not assess the specific sources of the ingroup and outgroup stress, it is not feasible to examine this possibility empirically. However, other studies (Meca et al., 2020) have found that invalidation of one’s identity across contexts may be most disruptive to a healthy self-concept. Future studies could explore whether the sources of identity invalidation (through in-group marginalization, such as identity threats or discrimination) matters vis-à-vis how in-group stressors are experienced and their effects on mental health outcomes (e.g., is a family member’s identity invalidation more harmful than a peer’s identity invalidation?).

The identification of these four profiles suggests differences in the specific types of cultural stress that are most present for youth. This finding can provide an opportunity for tailored interventions that directly address the clusters of cultural stressors that specific youth are most likely to experience. Some interventions (e.g., Zamora et al., 2019) use cuentos or vignettes that illustrate how cultural stressors are experienced, and typologies of cultural stressors can provide the content to create cuentos that explore clusters of cultural stress experienced. Importantly, the identification of intra-group marginalization as a prominent stressor underscores the need to address within-group, as well as outgroup, discrimination and racism within interventions designed to promote cultural assets and/or buffer the effects of cultural stressors.

### Demographic Differences

The profile solution varied across sites, with Low profile characterized by the most equal distribution of participants across Miami and Los Angeles, but with all other profiles composed primarily of Los Angeles participants (Sociopolitical and Language Brokering: 73.47%, Sociopolitical and In-group Identity Threats: 68.75%, Higher Stress: 76.47%). At the time of data collection, the political environment was most hostile in Los Angeles, where youth perceived and experienced distress as a result of the negative rhetoric surrounding U.S. immigration policies (Dunn et al., 2022). Previous studies have found that documentation status may buffer the impact of sociopolitical stressors. Although this study did not measure documentation status as a covariate, it is very likely that Los Angeles participants (who were predominantly Mexican) knew of individuals that were undocumented in comparison to Miami participants, who were predominantly Cuban (where Cubans were offered a pathway to documentation as long as they arrived in the United States prior to December 2016; Soto-Vásquez & Gonzalez, 2022). Additionally, Miami tends to be far more politically conservative compared to Los Angeles. These differences in nationality and political orientation between sites could explain why, at the time of data collection, more Los Angeles participants than Miami participants were classified into the higher-stress profiles. The present pattern of site differences also emphasizes that sociopolitical stress is likely to be experienced differently based on the youth’s political heritage, and that in incongruent social contexts (i.e. an administration that differs from one’s personal political views or issues), stress is more likely to be experienced (Morey et al., 2021).

Differences also emerged in participants’ nativity across profiles, with a predominance of U.S. born youth across all profiles. The data collection period coincided with the early closures within the COVID-19 pandemic that necessitated a shift toward virtual data collection, which may have reduced our ability to recruit foreign-born students. However, our sample is representative of the current proportion of US born to foreign born Hispanic/Latino youth in the US, as more than 75% of Hispanic youth in the United States are US-born (Pew Research Center, 2020). Lastly, sex differences emerged across the profiles, with females more likely than males to belong to the high stress profiles. This finding is in alignment with literature that has found females to be more susceptible to internalizing symptoms than males (Castilla-Puentes et al., 2022).

### Differences across Mental Health Indicators and Cultural Assets

Depressive symptoms, self-esteem, and adolescent stress scores differed significantly across profiles. Compared to the Low profile, the other three profiles were characterized by significantly higher levels of depressive symptoms and of adolescent stress, as well as lower self-esteem. These findings are consistent with previous literature identifying cultural stressors as risk factors for poor mental health outcomes (Bekteshi & Kang, 2020). More specifically, the Sociopolitical and Language Brokering profile was associated with higher adolescent stress means compared to the Sociopolitical and In-group Identity Threat profile and was associated with significantly lower heritage orientation scores compared to all the other profiles. Low levels of heritage orientation may reflect a lack of Spanish language fluency, which would render language brokering more stressful. Although a number of strengths (e.g., pride, advanced linguistic ability, increased interpersonal skills, confidence, and maturity; Guan et al., 2014) are associated with language brokering, the context (i.e., frequency of brokering, family acculturation gap, norms, subject- such as medical translation, and efficacy; (Zhang et al., 2020) in which language brokering occurs and the negative affect (i.e. feeling burdened) experienced as a result of language brokering may be associated with internalizing symptoms, including depression (Shen et al., 2020). In the present study, the language brokering subscale indexed the time commitment, responsibility, and burden experienced as a result of translating, reflecting largely negative or stressful appraisals of language brokering. Hence, in our study, the profile characterized by high language brokering stress is aligned with previous literature linking language brokering with higher adolescent stress and lower heritage orientation.

Ethnic identity has often been associated in the literature with positive mental health outcomes (e.g., associated with higher self-esteem and lower psychological distress; Forrest-Bank & Cuellar, 2018). Interestingly, in our study, profiles with higher cultural stress were associated with higher ethnic identity compared to the low stress profile. Perhaps participants with higher levels of ethnic identity are more aware and sensitized to the negative rhetoric and political issues that impact their ethnic group, and in a harsh political context (such as early 2020 when this data was collected), this identification with an openly minoritized and scapegoated group may have contributed to an increased perception of cultural stress (Meca et al., 2020). Alternatively, from a strength-based perspective, exposure to cultural stressors might have led to the development of higher ethnic identity as a coping mechanism. Ethnic identity leads to a positive assessment of one’s ethnic group, which could then buffer the effects of discrimination or xenophobia (Rivas-Drake et al., 2021). Further studies are needed to deconstruct the effects of ethnic identity as a cultural asset and to explore its relationship with cultural stressors.

### Strengths and Limitations

A multi-site study provides many strengths, including a more representative sample of migrants from different countries and different contexts of reception. In the present sample, Mexico, Cuba, Central American countries, and South American countries were represented. The sample was predominantly (almost 80%) second generation (U.S. born youth with at least one foreign born parent), which is representative of the U.S. Hispanic/Latino population. Another strength of this study is that it is the first study to analyze cultural stress profiles using socio-political climate, language brokering, in-group identity threats, and within-group discrimination as indicators.

A possible limitation, however, is that Los Angeles and Miami tend to be considered more favorable contexts of reception than cities with lower Hispanic/Latin populations and may, therefore, theoretically attenuate the effects of cultural stress on mental health. A different profile solution might have emerged in a different set of receiving contexts. Further, the sample sizes were unbalanced across sites, with the experiences of Los Angeles participants overrepresented. Additionally, the fact that our study was conducted during the COVID-19 pandemic may have affected participants’ responses to our survey items. Future studies, where recruitment occurs in person after the end of the pandemic, may yield different findings than those obtained in the present study.

There are both strengths and limitations associated with the heavily US-born sample recruited for the present study. The experiences and perceptions of US-born youth are likely overrepresented in the present results. Future research would benefit from over-recruiting foreign born youth so that their experiences are more strongly reflected in the findings. Another limitation is the small sample size of the high stress profile, which limits the generalizability and reliability of this profile. Additionally, political events at the time of data collection, such as national anti-immigrant sentiment, may impact individuals’ experiences of political stressors, discrimination, and context of reception. This political context may represent both an advantage and a limitation. As an advantage, the especially harsh political rhetoric toward Hispanic/Latino communities may exemplify the reception that these communities have experienced increasingly over the past decades (Dreby, 2015). As a limitation, this rhetoric may have constrained the profiles that were most likely to emerge (i.e., three of the four profiles were characterized by high levels of sociopolitical stress).

Lastly, as with most studies, self-selection bias and priming effects are a common limitation. These were mitigated by analyzing the distribution of demographic characteristics and careful wording of the study description to avoid biasing participant’s responses.

## Conclusion

Although previous studied have examined the harmful effects of cultural stressors on mental health, these studies have not accounted for the co-occurrence of cultural stressors to varying extents among different subgroups of youth in real-world settings. This study addressed this gap by methodologically identifying profiles of cultural stress. The present results suggest that a somewhat understudied set of cultural stressors – language brokering stress, within-group marginalization, ingroup identity threat, and negative sociopolitical climate – may manifest in different combinations among Hispanic/Latino adolescents in two major US cities. Compared to the Low profile, profiles characterized by elevated levels of cultural stress were associated with lower self-esteem and with greater depressive symptoms and general adolescent stress – but also with higher ethnic identity. These results suggest that, although cultural stress can be damaging for Hispanic/Latino adolescent mental health, these youth may also identify sources of resilience against these cultural stressors. The present results also exemplify the importance of current events vis-à-vis research on cultural stress among Hispanic/Latino adolescents. It is hoped that these results find their way into intervention and policy work, so that the well-being of Hispanic/Latino youth – a rapidly growing segment of the US population – can be promoted and protected.

## Footnotes

1 The terms “Hispanic” and “Latino” are used throughout this article to refer to people who identify as Latino, Latina, Latine, Latinx, and Hispanic. These terms have been chosen because they represent the most widely used term by participants (Rodriguez, 2019).

## References

[CR1] Almeida J, Biello KB, Pedraza F, Wintner S, Viruell-Fuentes E (2016). The association between anti-immigrant policies and perceived discrimination among Latinos in the US: a multilevel analysis. SSM - Population Health.

[CR2] Andresen EM, Malmgren JA, Carter WB, Patrick DL (1994). Screening for depression in well older adults: evaluation of a short form of the CES-D. American Journal of Preventive Medicine.

[CR3] Anniko MK, Boersma K, van Wijk NPL, Byrne D, Tillfors M (2018). Development of a Shortened Version of the Adolescent Stress Questionnaire (ASQ-S): construct validity and sex invariance in a large sample of Swedish adolescents. Scandinavian Journal of Child and Adolescent Psychiatry and Psychology.

[CR4] Asparouhov T, Muthén B (2021). Auxiliary variables in mixture modeling: using the BCH method in Mplus to estimate a distal outcome model and an arbitrary second model. Mplus Web Notes.

[CR72] Bekteshi V, Kang SW (2020). Contextualizing acculturative stress among Latino immigrants in the United States: A systematic review. Ethnicity & health.

[CR5] Cadenas, G. A., Cerezo, A., Carlos Chavez, F. L., Capielo Rosario, C., Torres, L., Suro, B., Fuentes, M., & Sanchez, D. (2022). The citizenship shield: Mediated and moderated links between immigration status, discrimination, food insecurity, and negative health outcomes for latinx immigrants during the COVID-19 pandemic. *Journal of Community Psychology*, 1–17. 10.1002/jcop.22831. 10.1002/jcop.22831 PMC9088249 35243656

[CR6] Castilla-Puentes, W. I., Castilla-Puentes, R., & Castilla-Puentes, S. (2022). *Mental Health in Hispanic/Latina/Latinx Women*. In Mental Health for Hispanic Communities: A Guide for Practitioners (pp. 205–214). Cham: Springer International Publishing.

[CR7] Castillo LG (2009). The role of intragroup marginalization in Latino college student adjustment. International Journal for the Advancement of Counselling.

[CR8] Choy B, Arunachalam K, S G, Taylor M, Lee A (2021). Systematic review: acculturation strategies and their impact on the mental health of migrant populations. Public Health in Practice.

[CR9] Cobb, C. L., Meca, A., Schwartz, S. J., Zea, M. C., Fernandez, C. A., & Sanders, G. L. (2019). Perceived discrimination and well-being among unauthorized hispanic immigrants: the moderating role of ethnic/racial group identity centrality. *Cultural Diversity and Ethnic Minority Psychology*, *25*(2), 280–287. 10.1037/cdp0000227 30284850

[CR10] Compas, B. E. (2013). Processes of risk and resilience during adolescence: linking contexts and individuals. In *Handbook of Adolescent Psychology* (2nd ed., vol. 1). 10.1002/9780471726746.ch9.

[CR11] Compas BE, Jaser SS, Dunbar JP, Watson KH, Bettis AH, Gruhn MA, Williams EK (2014). Coping and emotion regulation from childhood to early adulthood: Points of convergence and divergence. Australian Journal of Psychology.

[CR12] Davis AN, McGinley M, Carlo G, Schwartz SJ, Unger JB, Rosiers SED, Baezconde-Garbanati L, Lorenzo-Blanco EI, Soto D (2021). Examining discrimination and familism values as longitudinal predictors of prosocial behaviors among recent immigrant adolescents. International Journal of Behavioral Development.

[CR13] Dreby, J. (2015). *Everyday illegal: When policies undermine immigrant families*. University of California Press.

[CR14] DeMaris, A. (2004). Multiple Regression with Categorical Predictors: ANOVA and ANCOVA Models. In *Regression with social data: modeling continuous and limited response variables* (pp. 126–161). 10.1002/0471677566.ch4

[CR15] Díaz McConnell E (2022). “It could be 3 million, it could be 30 million”: Quantitative misperceptions about undocumented immigration and immigration attitudes in the Trump era. Latino Studies.

[CR69] Dunn D, Wray-Lake L, Plummer JA (2022). Youth are watching: Adolescents’ sociopolitical development in the Trump era. Child Development.

[CR65] Enriquez LE, Morales Hernandez M, Ro A (2018). Deconstructing immigrant illegality: A mixed-methods investigation of stress and health among undocumented college students. Race and Social Problems.

[CR63] Fandl KJ (2017). Cuban migration to the United States in a post-normalized relations World. Minnesota Journal of International Law.

[CR16] Ferguson SL, G. Moore EW, Hull DM (2020). Finding latent groups in observed data: A primer on latent profile analysis in Mplus for applied researchers. International Journal of Behavioral Development.

[CR18] Forrest-Bank SS, Cuellar MJ (2018). The mediating effects of ethnic identity on the relationships between racial microaggression and psychological well-being. Social Work Research.

[CR19] Griffin, A. (2017). Adolescent neurological development and implications for health and well-being. *Healthcare*, *5*(4). 10.3390/healthcare5040062 10.3390/healthcare5040062 PMC5746696 28961184

[CR20] Guan SSA, Greenfield PM, Orellana MF (2014). Translating into understanding: language brokering and prosocial development in emerging adults from immigrant families. Journal of Adolescent Research.

[CR21] Hill TD, Needham BL (2013). Rethinking gender and mental health: a critical analysis of three propositions. Social Science and Medicine.

[CR22] Howard MC, Hoffman ME (2018). Variable-centered, person-centered, and person-specific approaches: where theory meets the method. Organizational Research Methods.

[CR23] Johnson AE, Perry NB, Hostinar CE, Gunnar MR (2019). Cognitive–affective strategies and cortisol stress reactivity in children and adolescents: normative development and effects of early life stress. Developmental Psychobiology.

[CR24] Kam JA, Lazarevic V (2014). The stressful (and not so stressful) nature of language brokering: identifying when brokering functions as a cultural stressor for Latino immigrant children in early adolescence. Journal of Youth and Adolescence.

[CR25] Kim, S. Y., Wen, W., Chen, S., Yan, J., Song, J., Zhang, M., & Zeiders, K. H. (2022). Mexican‐origin youths’ language brokering for fathers and mothers: daily experiences and youths’ diurnal cortisol slopes. *Child Development*, 1–15. 10.1111/cdev.13768 10.1111/cdev.13768 PMC10371207 35397115

[CR26] Krogstad, J. M. (2021). *Most Cuban American voters identify as Republican in 2020*. Pew Research Center. https://www.pewresearch.org/short-reads/2020/10/02/most-cuban-american-voters-identify-as-republican-in-2020/

[CR27] Lazarevic, V., Guan, S. S. A., & Weisskirch, R. S. (2022). Experiences of discrimination and language brokering: exploring risks and protective factors. *Cultural Diversity and Ethnic Minority Psychology*. 10.1037/cdp0000532 10.1037/cdp0000532 35201793

[CR28] Lazarus RS, Folkman S (1984). Stress, appraisal, and coping.

[CR74] Lopez, M. H., Krogstad, J. M., & Flores, A. (2020). Key facts about young latinos, one of the nation’s fastest-growing populations. Pew Research Center. https://www.pewresearch.org/short-reads/2018/09/13/key-facts-about-young-latinos/.

[CR29] Lorenzo-Blanco EI, Unger JB, Baezconde-Garbanati L, Ritt-Olson A, Soto D (2012). Acculturation, enculturation, and symptoms of depression in Hispanic youth: the roles of gender, Hispanic cultural values, and family functioning. Journal of Youth and Adolescence.

[CR30] Meca A, Gonzales-Backen M, Davis R, Rodil J, Soto D, Unger JB (2020). Discrimination and ethnic identity: establishing directionality among Latino/a youth. Developmental Psychology.

[CR31] Meca A, Webb T, Cowan I, Moulder A, Schwartz S, Szabó A, Ward C (2022). Effects of cultural stress on identity development and depression among Hispanic college students. Identity.

[CR32] McCord AL, Draucker CB, Bigatti S (2019). Cultural stressors and depressive symptoms in Latino/a adolescents: an integrative review. Journal of the American Psychiatric Nurses Association.

[CR33] Montero-Zamora, P., Salas-Wright, C. P., Maldonado-Molina, M. M., Brown, E. C., Vos, S. R., Garcia, M. F.,… & Schwartz, S. J. (2023a). Hurricane stress, cultural stress, and mental health among hurricane Maria migrants in the US mainland. *American Journal of Orthopsychiatry*. 10.1037/ort0000669. 10.1037/ort0000669 PMC11977423 36802364

[CR73] Montero-Zamora, P., Vos, S. R., Unger, J. B., Zeledon, I., Lee, R., Soto, D. W., ... & Schwartz, S. J. (2023b). Perceived negative political climate among Hispanic/Latino adolescents before and after the 2020 US presidential election: Associations with internalizing symptoms and substance use. International Journal of Intercultural Relations, *94*, 101790. 10.1016/j.ijintrel.2023.101790 PMC10121197 37091741

[CR71] Morey BN, García SJ, Nieri T, Bruckner TA, Link BG (2021). Symbolic disempowerment and Donald Trump’s 2016 presidential election: Mental health responses among Latinx and white populations. Social Science & Medicine.

[CR34] Muthén LK, Muthén BO (2018). Mplus user’s guide, version 8.3.

[CR35] Nylund K, Asparouhov T, Muthen B (2007). Deciding on the number of classes in latent class analysis and growth mixture modeling: a Monte Carlo simulation study. Structural Equation Modeling.

[CR64] Passel, J. S., & Cohn, D. (2020). Most U.S. unauthorized immigrants live in just 20 metro areas. Pew Research Center. https://www.pewresearch.org/short-reads/2019/03/11/us-metro-areas-unauthorized-immigrants/.

[CR36] Perreira KM, Marchante AN, Schwartz SJ, Isasi CR, Carnethon MR, Corliss HL, Kaplan RC, Santisteban DA, Vidot DC, Van Horn L, Delamater AM (2019). Stress and resilience: key correlates of mental health and substance use in the Hispanic Community Health Study of Latino Youth. Journal of Immigrant and Minority Health.

[CR37] Phinney JS (1992). The multigroup ethnic identity measure (1992).pdf. Journal of Adolescent Research.

[CR38] Rivas-Drake D, Pinetta BJ, Juang LP, Agi A (2021). Ethnic-racial identity as a source of resilience and resistance in the context of racism and xenophobia. Review of General Psychology.

[CR39] Roche KM, Walsdorf AA, Jordan LS, Falusi OO (2021). The contemporary anti-immigrant environment and Latin American-origin adolescents’ perceived futures: a phenomenographic content analysis. Journal of Child and Family Studies.

[CR40] Roche KM, White RMB, Rivera MI, Safa MD, Newman D, Falusi O (2020). Recent immigration actions and news and the adjustment of U.S. Latino/a adolescents. Cultural Diversity and Ethnic Minority Psychology.

[CR66] Rodriguez C (2016). Experiencing ‘illegality’as a family? Immigration enforcement, social policies, and discourses targeting Mexican mixed‐status families. Sociology Compass.

[CR41] Rodriguez, A. (2019). ’Latinx’ explained: a history of the controversial word and how to pronounce it. USA Today. https://www.usatoday.com/story/news/nation/2019/06/29/latina-latino-latinx-hispanicwhat-do-they-mean/1596501001/.

[CR42] Rosenberg, M. (1979). Conceiving the Self (Book). In *Journal of Personality Assessment* (Vol. 45, Issue 4). 10.1207/s15327752jpa4504_23

[CR43] Salas-Wright CP, Vaughn MG, Goings TC, Cobb CL, Cohen M, Montero-Zamora P, Eschmann R, John R, Andrade P, Oliveros K, Rodríguez J, Maldonado-Molina MM, Schwartz SJ (2020). Toward a typology of transnational communication among Venezuelan immigrant youth: implications for behavioral health. Journal of Immigrant and Minority Health.

[CR44] Salas-Wright CP, Maldonado-Molina MM, Brown EC, Bates M, Rodríguez J, García MF, Schwartz SJ (2021). Cultural stress theory in the context of family crisis migration: implications for behavioral health with illustrations from the Adelante Boricua Study. American Journal of Criminal Justice.

[CR45] Schwartz SJ, Unger JB, Zamboanga BL, Szapocznik J (2010). Rethinking the concept of acculturation: implications for theory and research. American Psychologist.

[CR46] Schwartz SJ, Unger JB, Des Rosiers SE, Lorenzo-Blanco EI, Zamboanga BL, Huang S, Baezconde-Garbanati L, Villamar JA, Soto DW, Pattarroyo M, Szapocznik J (2014). Domains of acculturation and their effects on substance use and sexual behavior in recent hispanic immigrant adolescents. Prevention Science.

[CR47] Schwartz SJ, Unger JB, Zamboanga BL, Córdova D, Mason CA, Huang S, Baezconde-Garbanati L, Lorenzo-Blanco EI, Des Rosiers SE, Soto DW, Villamar JA, Pattarroyo M, Lizzi KM, Szapocznik J (2015). Developmental trajectories of acculturation: links with family functioning and mental health in recent-immigrant hispanic adolescents. Child Development.

[CR48] Shen Y, Seo E, Walt DC, Kim SY (2020). Stress of language brokering and Mexican American adolescents’ adjustment: the role of cumulative risk. Journal of Early Adolescence.

[CR49] Simmons, A. B. L., & Nelson, D. L. (2012). Eustress at work: extending the holistic stress model. In *Sage Books Positive Organizational Behavior* (pp. 2–17). 10.4135/9781446212752.

[CR50] Smith TB, Silva L (2011). Ethnic identity and personal well-being of people of color: a meta-analysis. Journal of Counseling Psychology.

[CR67] Smokowski, P. R., & Bacallao, M. (2011). Worlds apart: Bicultural identity development in Latino adolescents. In Acculturation: Implications for Individuals, Families and Societies (pp. 133–149). Nova Science Publishers, Inc.

[CR70] Soto-Vásquez AD, Gonzalez E (2022). “Not a Monolith!” Media Narratives of the Latina/o/x Vote after the 2020 US Election. Howard Journal of Communications.

[CR51] Spurk D, Hirschi A, Wang M, Valero D, Kauffeld S (2020). Latent profile analysis: a review and “how to” guide of its application within vocational behavior research. Journal of Vocational Behavior.

[CR52] Swann W. B. Jr., & Buhrmester, M. D. (2012). Self-verification: The search for coherence. In Leary M. R. & Tangney, J. P. (Eds.), Handbook of self and identity (pp. 405–424). The Guilford Press.

[CR53] Szapocznik J, Kurtines WM, Fernandez T (1980). Bicultural involvement and adjustment in Hispanic-American youths. International Journal of Intercultural Relations.

[CR54] Thornhill, C. W., Castillo, L. G., Piña-Watson, B., Manzo, G. & Cano, M. Á. (2021). Mental health among Latinx emerging adults: examining the role of familial accusations of assimilation and ethnic identity. *Journal of Clinical Psychology, 2021*, 892–912. 10.1002/jclp.23271. 10.1002/jclp.23271 PMC9035025 34726784

[CR55] Torres JM, Young MED (2016). A life-course perspective on legal status stratification and health. SSM - Population Health.

[CR56] Unger JB, Ritt-Olson A, Teran L, Huang T, Hoffman BR, Palmer P (2002). Cultural values and substance use in a multiethnic sample of California adolescents. Addiction Research and Theory.

[CR57] Vatcheva K, Lee M (2016). Multicollinearity in regression analyses conducted in epidemiologic studies. Epidemiology: Open Access.

[CR58] Vos SR, Hee C, Alvarez VC, Meca A, Unger JB, Brown EC, Zeledon I, Soto D, Schwartz SJ (2021). International Journal of Intercultural Relations Cultural stress in the age of mass xenophobia: perspectives from Latin/o adolescents. International Journal of Intercultural Relations.

[CR59] Weisskirch, R. S. (2017). A developmental perspective on language brokering. In *Language Brokering in Immigrant Families: Theories and Contexts* (pp. 7–25). Routledge/Taylor & Francis Group.

[CR68] Zamora AL, Curtis H, Lancaster L (2019). Promoting racial and ethnic identity: A school-based intervention to support Latino youth. Journal of Latinos and Education.

[CR60] Zeledon, I., Unger, J. B., West, A. E., Cruz, N., & Schwartz, S. J. (2022). Immigration and cultural stressors and their impact on mental health outcomes. In *Encyclopedia of Child and Adolescent Health* (pp. 1–12). Elsevier Inc. 10.1016/B978-0-12-818872-9.00197-7.

[CR61] Zhang M, Kim SY, Hou Y, Shen Y (2020). Parent–adolescent acculturation profiles and adolescent language brokering experiences in Mexican immigrant families. Journal of Youth and Adolescence.

[CR62] Zhen-Duan J, Jacquez F, Saez-Santiago E (2011). Boricua de Pura Cepa: ethnic identity, cultural stress and self- concept in Puerto Rican youth. Physiology & Behavior.

